# Assessment of the Chronic Toxicity and Interactions between Arsenic and Riverbed Biofilms

**DOI:** 10.3390/ijerph191912689

**Published:** 2022-10-04

**Authors:** María Teresa Barral, Diego Rodríguez-Iglesias, Diego Martiñá-Prieto, Remigio Paradelo

**Affiliations:** 1 Department of Soil Science and Agricultural Chemistry, University of Santiago de Compostela, 15782 Santiago de Compostela, Spain; 2 Cross-Research in Environmental Technologies (CRETUS), University of Santiago de Compostela, 15782 Santiago de Compostela, Spain

**Keywords:** ecotoxicity, arsenic speciation, pollution-induced community induced tolerance, fluorescence, algal pigments, microphytobenthos

## Abstract

The toxic effect of exposure to arsenic, As(V), at concentrations of 0 to 30 mg L^−1^, for 49 days, on epipsammic biofilms, was evaluated in a microcosm experiment. The growth and composition of biofilms developed on sediments containing As concentrations of 31 mg kg^−1^ and 85 mg kg^−1^ were compared, using photosynthetic parameters and Live/Dead stains as end points. A toxic effect of arsenic could not be demonstrated; however, biofilm growth was higher over the sediment with higher arsenic concentrations, suggesting the development of pollution-induced community induced tolerance (PICT). Nevertheless, PICT was not observed after exposure to high arsenic concentration in the laboratory, as there were no differences in algal growth between the previous 0 and 30 mg L^−1^ systems exposed to new 30 mg As L^−1^ dissolution over 29 days. The algal composition was affected by the added arsenic, and brown algae were the most tolerant compared to green algae and cyanophyceae, as their percentage increased from 25 and 33% in the control samples to 57 and 47% in the samples with the highest added As concentration. In turn, the biofilm development influenced arsenic redistribution and speciation. Arsenic concentration in water decreased with time during the incubation experiment, retained by the sediment particles and the biofilm. In the biofilm, extracellular As was significantly higher (up to 11 times) than intracellular arsenic. As(V) was the predominant species in water and in the biofilm, but products of biotic transformation, namely As(III), DMA(V) and MMA(V), were also found in the solution and in the biofilm in some systems, demonstrating reduction and methylation by the organisms. As a conclusion, a toxic effect was not detected for the concentrations evaluated. Biofilms naturally exposed in the river system to high As concentrations acquire pollution-induced tolerance; however, tolerance was not acquired by exposure to 30 mg L^−1^ for 29 days in the laboratory.

## 1. Introduction

Biofilms are populations of microorganisms (algae, bacteria and fungi) attached to surfaces and enclosed in a matrix of extracellular polymeric substances (EPSs), which form a protective layer for the cells and serve as carbon and energy reserves when nutrients are scarce [1,2,3]. In aquatic environments, biofilms are located at the interface between the overlying water and the substratum, which can be sediment grains, rocks or plants, named epipsammic, epilithic or epiphytic biofilms, respectively.

Biofilms can retain metals and metalloids from the overlying water through a variety of mechanisms, including (bio-)sorption, precipitation as sulfides or phosphates and microbial reductive precipitation [4]. In turn, pollutants may affect the biomass, composition and functionality of biofilm communities [5]. Therefore, biofilms are considered good bioindicators of water quality [6,7] and can be regarded as early warning systems for the detection of toxicant effects on aquatic systems.

The potential for arsenic retention in aquatic biofilms has been previously reported [8,9,10]. In systems where P and As occur simultaneously, biofilms favor As retention by counteracting the effect of phosphate, which otherwise would prevent arsenate adsorption due to their chemical similarities [11]. Biofilms not only retain, but also modify, arsenic speciation and mobility in aqueous systems [12,13]. In fluvial ecosystems, it has been demonstrated that the microorganisms forming the biofilm incorporate the dominant inorganic As forms (iAs) and transform it through metabolic or detoxifying processes, which has a big impact on the ecotoxicological behavior of arsenic [14,15].

The interaction between As and biofilm is mutual, since exposure to As may alter the physiology and structure of biofilms, leading to changes in ecosystem function and trophic relations [15]. However, to date, it has not been clearly established what As concentrations effectively alter the biomass, structure and functions of fluvial biofilms, and the range of values suspected to have ecotoxic effects covers several orders of magnitude. This question is difficult to solve, because toxicity is influenced by As species and other factors, such as the adsorption to mineral particles, active biological surfaces, biotransformation and/or nutrient availability [16,17,18,19].

Another aspect to be considered, regarding the vulnerability of the fluvial biofilm to As toxicity, is the development of pollution-induced community tolerance (PICT) due to chronic exposure of a stressed community to contaminants, which may exert a selection pressure on natural communities, eliminating sensitive species and, thereby, increasing their tolerance [20]. However, to our knowledge, the pollution-induced tolerance in fluvial biofilms exposed to arsenic has been little explored.

With the aim of advancing knowledge of the mutual interactions between As and epipsammic biofilms and their environmental and ecotoxicological consequences, this work focused on the effect of As on biofilm growth and possible PICT development in biofilms exposed to this element and, conversely, on the effects of biofilms on arsenic retention and speciation.

This study was conducted with water and sediment samples taken from the Anllóns River, Galicia (NW Spain), where high As concentrations are found in the riverbed sediments, attributed to geogenic enrichment exacerbated by mining activities [21]. Arsenopyrite mineralization in hydrothermal quartz veins [22] are associated with gold ores that were exploited during the Roman Empire and then from 1895 until 1910, with intermittent activity since then. Arsenic concentrations in mineralized zones with semi-massive arsenopyrite can reach up to 10%, whereas in the superficial soil horizons arsenic contents of 4000 mg kg^−1^ have been observed [23]. In the riverbed sediments, As concentrations of up to 264 mg kg^−1^ were detected downstream of the mineralized area to the river mouth [21,24]. The release of As from the Anllóns basin to the coastal areas, where intense shellfish harvesting takes place, is relevant, as it was estimated that the river exports 460 kg y^−1^ of dissolved arsenic to its estuary [25]. Most As in the sediments of the Anllóns River is found in low-mobility forms, associated with Fe-oxides and in the residual phase [21,26]. Nevertheless, this favorable condition does not have to be permanent, because changes in environmental conditions affect As solubility. If mobilized, these high concentrations of As may result in serious environmental damage (timed chemical bomb). Thus, for instance, it was observed that As mobility in these sediments was strongly dependent on pH; it occurred simultaneously with the dissolution of oxides and hydroxides of Fe and Al at acidic pH, and with that of organic matter at alkaline pH [26]. Increased release of As from contaminated sediments was observed with increasing water: sediment ratios, suggesting a risk of mobilization during high-flow resuspension events [24]. Arsenic mobility was also favored in high ionic strength conditions, typical of estuarine environments where the mixture of fresh and marine waters occurs [26], and in the presence of high concentrations of phosphate, which may come from wastewaters and fertilizers eroded or leached from the soils of the basin [27,28]. The Anllóns sediment surfaces are commonly covered by epipsammic biofilms [29], mainly constituted of Bacillariophyceae [30], so their effect on As mobility must also be considered. In a previous study [13] it was observed that the retention of As(V) from the water column was higher in sediments from the Anllóns covered with biofilm than in those devoid of biofilm and, conversely, the biofilm inhibited the release of As(V) from As-polluted sediments to the water column. The biofilm also affected As speciation, by inhibiting the reduction of As(V) to As(III) and increasing the concentration of organic species, such as monomethylarsenate (MMA(V)) and dimethylarsenate (DMA(V)).

The hypotheses of this study are the following: (1) the biofilm affects As retention and transformation; (2) the presence of As in water may have a toxic effect on sediment biofilm, limiting its growth; (3) the biofilm grown on As-contaminated sediments may develop acquired tolerance (PICT) to this element; and (4) biofilms exposed in the laboratory to high added As concentrations may also acquire tolerance.

To test these hypotheses, experiments were carried out at a microcosm level to compare the biofilm growth on As-polluted and unpolluted riverbed sediments, which were exposed to different added As concentrations. Photosynthetic parameters were the main parameters used as end points. The evolution of total As concentration and its speciation in the overlying water, as well as its accumulation and distribution in the biofilm, were analyzed. The potential development of pollution-induced community induced tolerance by the biofilms due to exposure to natural or added As was also evaluated.

## 2. Materials and Methods

### 2.1. Sampling and Characteristics of Water and Sediments

Two sites along the Anllóns river course were selected for sampling sediments and water:

- Eguas (43°13′24.26″ N, 8°45′44.61″ W): not affected by mining activities, so As concentration in sediments was expected to be low.
- Xavarido (43°13′48.82″ N, 8°49′54.29″ W): located 8 km downstream Eguas, just a little downstream from the area affected by mining, and known to have high As concentrations.

At each point, river water was taken, and a complex sediment sample was collected with a small plastic shovel, mixing 4 to 6 subsamples from the top 5 cm at various points at each site. Sediment samples were taken to the laboratory in hermetic plastic containers, covered by a water layer from the same site to prevent oxidation.

The characteristics of the water samples are shown in Table 1. Both sites presented similar values of pH, EC, alkalinity, total N and cations. Total P concentration was higher in Eguas than in Xavarido, although lower than the maximum acceptable concentration to avoid accelerated eutrophication or to promote algal blooms, fixed at 0.1 mg L^−1^ [31].

Xavarido showed higher dissolved arsenic (1.33 µg L^−1^) than Eguas (0.85 µg L^−1^). Both concentrations were well below the limits set by U.S. EPA [32] for As concentration in freshwaters to protect aquatic organisms, as Criterion Maximum Concentration (CMC) is set at 340 µg L^−1^, while the Criterion Continuous Concentrations (CCC) for chronic exposure is 150 µg L^−1^. A much lower concentration was established by the World Health Organization for drinking water (10 µg L^−1^) [33].

The characteristics of the sediments are shown in Table 2. The granulometry was finer in Eguas, with 59% of the particles < 0.05 mm, whereas in Xavarido this fraction represented less than 2%. Eguas presented much higher C contents (9.19%) than Xavarido (0.44%), and a higher carbon to nitrogen ratio, that suggested an allochthonous origin (terrestrial) for the first (C/N > 12) and an autochthonous origin (algae and phytoplankton) for the second (C/N < 12) [34]. Arsenic concentration in the sediment from Eguas was 31 mg kg^−1^, while 85 mg kg^−1^ were found in Xavarido; this concentration exceeded the general reference As levels for soils in Galicia, fixed at 50 mg kg^−1^ [35], and was higher than the thresholds of the European Water Framework Directive 2000/60/EC [36] for suspended matter and river sediment (40 mg kg^−1^).

### 2.2. Experimental

#### 2.2.1. Chronic Toxicity Experiment

Sediment samples of particle size < 2 mm taken from Xavarido and Eguas were incubated for 42 days under controlled light conditions in an incubation chamber equipped with two bulbs (OSRAM DULUX^®®^, cool daylight, 28 µmol photon m^−2^ s^−1^, OSRAM GmbH, Munich, Germany) in 12:12 h day: night cycles. Temperature was maintained at 20–22 °C and air-supply conditions were 1.5 L min^−1^ pump flow, 2 h in the morning and 2 h in the afternoon.

The effect of As concentration on biofilm growth was assayed using six concentrations: 0 (control), 0.3, 1, 3, 10 and 30 mg L^−1^. For each sediment, As solutions were prepared in the river water taken from the same sampling site, filtered by 0.45 µm pore size (Puradisc 25 Syringe Filter, 0.45 µm PES membrane, Whatman, GE Healthcare Life Sciences, Dorset, UK), to which di-sodium hydrogen arsenate 7-hydrate (Na_2_HAsO_4_.7H_2_O, Panreac Química Sau, Barcelona, Spain) was added as As source. The sediment: water ratio was 1:4 (62.5 g of sediment and 250 mL of water). The experiments were carried out in triplicate, and so, for each location, 18 flasks were incubated.

During the experiment, the growth of photosynthetic microorganisms in the biofilm was monitored at 8 specific days using a Phyto-PAM device. Simultaneously, 2 mL aliquots of the overlying water were removed from each flask to determine total As concentration and speciation. At the end of the experiment, Live/Dead stain was applied to the biofilm to determine the number of live and dead bacteria, and the distribution and speciation of As in extracellular and intracellular compartments of the biofilm was evaluated, and total As associated to EPS was also measured.

#### 2.2.2. Natural Tolerance Evaluation

This experiment was carried out using the triplicate systems of control (0 mg L^−1^) and the highest As concentration (30 mg L^−1^) from the previous experiment, for Eguas and Xavarido sediments. First, the overlying solutions were carefully removed from the flasks and then 250 mL of new 30 mg L^−1^ As dissolution were added, followed by incubation for 29 days under the same conditions of light, temperature and air supply described in the first experiment. The evolution of the autotrophic component of the biofilm was monitored with Phyto-PAM at five specific days. Simultaneously, total As concentration was determined in 2 mL aliquots of the overlying water of each flask.

### 2.3. Analytical Methods

#### 2.3.1. Biofilm Growth and Characteristics

##### Phytoplankton Pulse-Amplitude-Modulated (Phyto-PAM) Measurements

The growth of photosynthetic organisms and the relative abundance of green and brown algae, and cyanobacteria in the biofilm was monitored with a Phyto-PAM fluorometer analyzer (Phyto-EDF, Phytoplankton Analyzer, Heinz Walz GMBH, Effeltrich, Germany). For each flask, eight saturation pulses were applied at different points on the sediment surface to obtain a representative average measure of the parameters. First, the minimum fluorescence yield (Fo) was given by the fluorometer in dark adapted samples and then a saturation pulse was applied to obtain the maximum PSII quantum yield (Ymax). After 15 min of light adaptation, a saturation pulse of actinic light was applied to the samples to obtain the effective PSII quantum yield (Yeff) and photochemical quenching (Qp). Fo provides an estimation of algal biomass, Ymax is a measurement of the photosynthetic capacity of the community, Yeff is a measurement of the community photosynthetic efficiency and Qp corresponds to the percentage of the energy used as fuel in the photosynthesis [37].

##### Live/Dead Analyses

An estimation of the live and dead bacterial proportion was obtained by staining, following the protocol described in Romaní et al. [38] and Proia [3]. Two replicates of the control, 3 and 30 mg As L^−1^ samples from Experiment 1 were randomly selected for the analysis. Then, 5 mL of the biofilm-enriched surface were pipetted from various points of the sediment surface and collected in Falcon tubes that were submitted to sonication in cold water for one minute at 35 W and 35 kHz frequency (Sonics Vibra-Cell^TM^ CV33, Sonics & Materials, Inc., Newtown, CT, USA). The suspensions were allowed to stand for 1–2 min, and, then, 3 mL of the biofilm-enriched supernatant were transferred to Falcon tubes for the Live/Dead staining, using the LIVE/DEAD^®®^ BackLight™ Bacterial Viability Kit (Molecular Probes, Inc. Invitrogen detection technologies, Leiden, The Netherlands), which stains only bacteria, the main heterotrophic organisms in the biofilm. A dye mixture was prepared by mixing the two components included in the Live/Dead kit in 1:1 ratio: 300 µL solution of 3.34 mM SYTO 9 dye (component A) in dimethylsulfoxide (DMSO), and 300 µL of 20 mM propidium iodide (component B) in DMSO. Then 1 µL of the dye mixture was added to each mL of the sample suspensions, thoroughly mixed and incubated at room temperature in darkness. The filters were placed on slides and Confocal Laser Scanning Microscope (LEICA TCS-SP2, LEICA Microsystems Heidelberg GmbH, Mannheim, Germany) was used to obtain a representative counting of live bacteria (shown in green) and dead bacteria (red), using an image processing software (ImageJ, Image Processing and Analysis in Java, version 1.48).

#### 2.3.2. Arsenic

##### Arsenic in Water

The total As concentration was determined by ICP-MS (Varian 820MS, Varian Medical Systems, Inc., Palo Alto, CA, USA), equipped with collision reaction interface (CRI) technology to reduce polyatomic interferences. Relative standard deviations for total As analysis by ICP-MS were <3%. As speciation in water was determined by HPLC-ICP-MS (Varian Prostar 230 HPLC, Varian Medical Systems, Inc., Palo Alto, CA, USA) equipped with a guard column and an anion exchange column Hamilton PRP-X100 (4.1 × 250 mm and 10 µm). Separation of the five arsenic species was performed using a 13 min gradient LC method with 12.5 mM and 30 mM (pH = 9) (NH_4_)_2_CO_3_ as mobile phase, a flow rate of 1 mL min^−1^ and an injection volume of 50 µL. The following species were determined: inorganic As(V) and As(III), and organic species dimethylarsenate (DMA(V)), monomethylarsenate (MMA(V)) and arsenobetaine (As-Bet). The detection limits under the experimental conditions were 2.8, 4.1, 2.9, 4.6 and 2.5 ng L^−1^ for As(V), As(III), MMA(V), DMA(V) and As-Bet, respectively. The certified reference material EnvironMAT-Drinking Water HIGH EP-H-1 (Catalog number: 140-025-032, SCP Science, Quebec H9X 4B6, QC, Canada) was used for quality control.

##### Arsenic in Biofilm

Five ml of biofilm-enriched surface were pipetted from each replicate by sampling different points of the sediment surface, transferred to Falcon tubes and sonicated for 2 min. Then, 3 mL supernatant were filtered through a 0.22 µm filter and washed with 10 mL river water to remove arsenic not adsorbed to cells. A sequential extraction was performed, following the procedure by Levy and collaborators [16], consisting in an extraction with KH_2_PO_4_/K_2_HPO_4_ (pH = 5.95) buffer solution for extracellular As, followed by digestion with 25% (*v*/*v*) HNO_3_ for 30 min in a microwave. Arsenic concentrations and chemical species were quantified by ICP-MS (Varian 820MS, Varian Medical Systems, Inc., Palo Alto, CA, USA) and by HPLC-ICP-MS (Varian Prostar 230 HPLC-Varian 820 MS, Varian Medical Systems, Inc., Palo Alto, CA, USA), respectively.

##### Arsenic in EPS

The concentration of EPS was estimated by the carbohydrates content, as they are the most abundant components in EPS matrices. For this, 500 mg of biofilm were collected from samples exposed to 0.3, 1, 3 and 10 mg L^−1^ of As and extracted in 5 mL 100 mM Na_2_EDTA (di-sodium ethylenediaminetetraacetic acid, PANREAC, Barcelona, Spain), vortex-mixed and incubated for 15 min at 20 °C [39]. A 2 mL aliquot was employed for total carbohydrate determination by the phenol-sulfuric acid assay [40]. Another 2 mL of supernatant were filtered by 0.45 µm and used for the quantification of total As in EPS by ICP-MS.

### 2.4. Statistics

Repeated measures ANOVA was employed to detect differences due to time and arsenic concentrations in the biofilm growth and other photosynthetic parameters. One-way ANOVA was carried out to find significant differences in Live/Dead, EPS concentration, and extracellular and intracellular arsenic analyses at the end of the experiment. Post hoc Tukey’s test was applied to check significant differences in all cases (*p* < 0.05; α = 0.05). All statistical analyses were performed using the SPSS Statistics package version 19.

## 3. Results

### 3.1. Effect of As Concentration on Biofilm Growth

**Figure 1:**
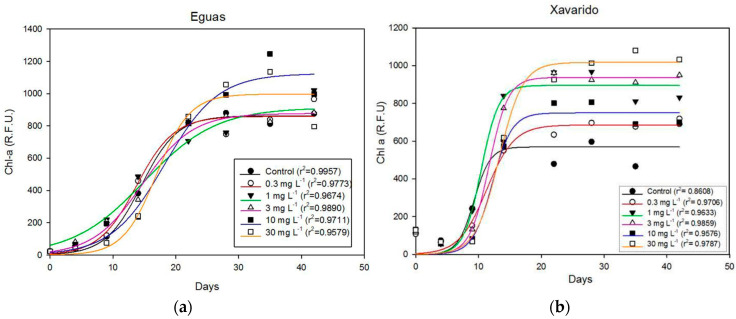
Biofilm growth on sediments exposed to increasing As concentrations, measured by Chl-a fluorescence (F_0_) expressed in Relative Fluoresce Units (R.F.U.). (**a**) Eguas systems. (**b**) Xavarido systems. Data were obtained at day 4, 9, 14, 22, 28, 35 and 42.

Figure 1, where minimum chlorophyll fluorescence yield (F_0_) was plotted versus time, summarizes the biofilm growth in Experiment 1, where sediments from Eguas and Xavarido were exposed for 42 days to different added As concentrations to detect chronic toxicity. F_0_ provides an indirect measure of algal biomass and is, thus, an indicator of biofilm growth. A sigmoidal growth was observed for all the As concentrations. For Eguas samples, there were significant differences (*p* < 0.05) in F_0_ with time until day 22, when a stationary phase began, but there were no significant differences between the different concentrations tested, indicating that there was not a toxic effect of As on the growth of the autotrophic components of the biofilm, at the concentrations essayed. For the Xavarido samples, a significant effect of time on growth was also observed, the stationary phase beginning at day 14. Significant differences due to As concentration were observed in F_0_ at specific times. Thus, at day 9, F_0_ values were significantly lower for the highest As concentrations (10–30 mg L^−1^) in comparison with the lowest As concentrations (0, 0.3, 1 and 3 mg L^−1^), but at day 35 the behavior was reversed, and the growth of biofilms exposed to 30 mg L^−1^ As was significantly higher than that of the control systems.

Therefore, from these results it could not be stated that the tested As concentrations had a toxic effect on biofilm growth, in the conditions essayed. To compare the growth in Eguas and Xavarido, it must be considered that Phyto-PAM measurements were made using different gains to achieve the necessary sensibility for each site. According to calibration values, F_0_ of Xavarido must be multiplied by two, so that the biofilm growth deduced from F_0_ was approximately twice that of Eguas. Since the growth of algae in both controls was similar and that of As-added samples higher in Xavarido, this behavior pointed to a greater tolerance of the biofilm from this site, attributed to its exposure to environmental As.

Other photosynthetic parameters were determined during biofilm growth. In Table 3 the values for time, in terms of days 0, 9 and 42, are shown (the whole data set for the seven dates of analyses is shown in the Supplementary Material, Table S1). The maximal quantum yield (Y_max_), indicative of the photosynthetic capacity of the community, did not differ with arsenic concentrations, except for the 10 and 30 mg L^−1^ concentrations at day 9 in Xavarido, for which significantly lower values were found, as was F_0_. The effective quantum yield (Y_eff_) only showed significantly lower values for 10 and 30 mg As L^−1^, at days 9 and 14, in Eguas samples. Finally, no significant differences were observed for the photochemical quenching (qP) that represents the energy used for photosynthesis.

**Figure 2:**
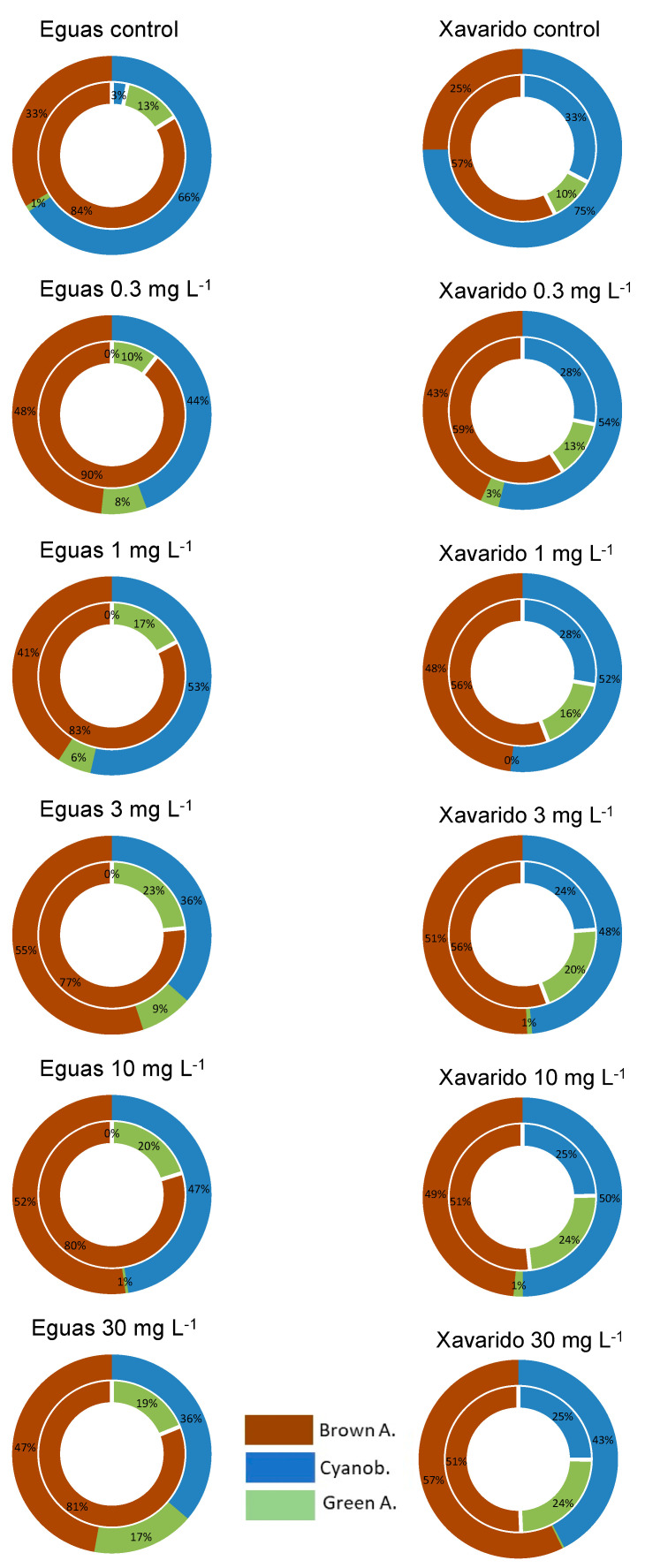
Algae distribution in 0 mg As L^−1^ (control) and 30 mg As L^−1^ systems from Eguas and Xavarido. Internal circle corresponds to day 0 and external circle to day 42 of the experiment. Brown A.: Brown algae; Cyanob.: Cyanobacteria; Green A.: Green algae.

From the excitation of the chlorophyll fluorescence at different wavelengths (440, 520, 645 nm) information was obtained on the relative contribution of differently pigmented organisms (green algae, brown algae and cyanobacteria, respectively) to the whole community, which was estimated by the signal at 665 nm, corresponding to chlorophyll-a (Figure 2) (the whole data set is shown in the Supplementary Material, Table S2).

In Eguas systems, the control systems had an initial composition of 84% brown algae (mainly represented by diatoms), 13% green algae and 3% cyanobacteria; this latter group increased during the incubation to become the most abundant (66%) at the end of the experiment. In the samples exposed to added As, cyanobacteria also increased with time up to day 14–22 and then decreased, and the opposite was observed for brown algae, so that a similar, or slightly higher, percentage of brown algae was observed at the end of the experiment, in comparison with cyanobacteria. Green algae showed, in all cases, a great variability, which can be partly attributed to the fact that they might detach from the biofilm, being filamentous forms, and interfere in the in-situ measurement of fluorescence. In the Xavarido systems, although brown algae were the most abundant in the control systems, there was an important presence of cyanobacteria (33%) at the beginning of the incubation. Then, the proportion of brown algae increased and dominated in the biofilm, until day 14; from that date, the cyanobacteria percentage progressively increased and became the dominant algal group at the end of the experiment. In the Xavarido systems incorporating As, cyanobacteria diminished with time, while brown algae increased, reaching similar values at the end of incubation, without the presence of green algae.

From these data, the clearest trend that could be deduced, regarding the possible effect of the addition of As on algae composition of the biofilm, was that brown algae were favored, were are more tolerant, in comparison with green algae and cyanobacteria, as the percentages of these two latter groups were consistently lower in As-added samples than in control samples.

**Figure 3:**
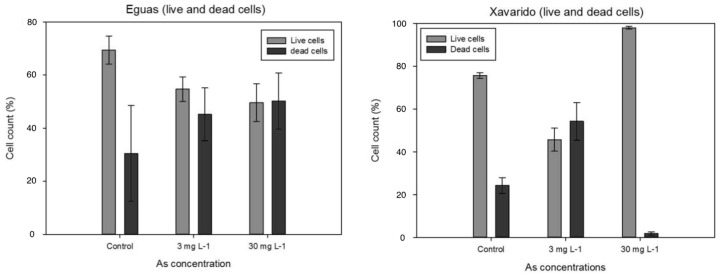
Live/Dead count for Eguas and Xavarido (mean ± SD, *n* = 3) exposed to different added As concentrations.

Live/Dead analyses showed (Figure 3) that the count of live bacterial cells in Eguas was significantly lower in the samples exposed to 3 and 30 mg L^−1^ than in control samples, but no significant differences were observed in dead bacteria. In Xavarido, only the system with 3 mg L^−1^ had significantly lower counts of live cells, but all the systems were different in the count of dead cells, with the highest value for 3 mg L^−1^ and the lowest for 30 mg L^−1^.

### 3.2. Arsenic in Water and Biofilm

**Figure 4:**
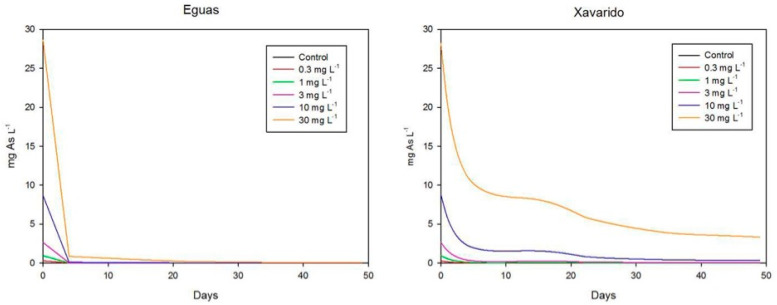
Evolution of total arsenic concentration in water during the first incubation experiment.

The variation in time of arsenic concentrations in the overlying water, in Experiment 1, is shown in Figure 4. In Eguas systems with added As, the As was rapidly removed from water, reaching in all cases retention values higher than 97%. In Xavarido, the As concentration reduced more progressively, reaching final reduction values > 96% for 0 to 10 mg L^−1^, and 88% for 30 mg L^−1^.

**Figure 5:**
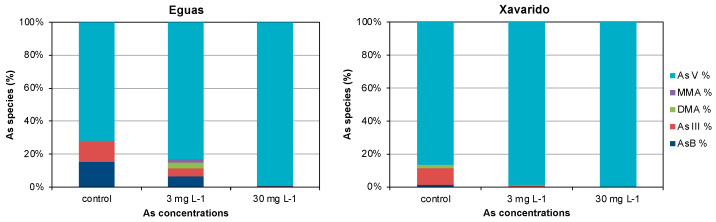
Speciation of As in control, 3 and 30 mg As L^−1^ at day 42 from Eguas and Xavarido systems.

Arsenic speciation in the overlying water of 0 (control), 3 and 30 mg L^−1^ systems of Eguas and Xavarido was analyzed at the end of incubation (Figure 5). It is noteworthy that, while the only species present in the river water at both sites was As(V), the species DMA(V), As-Bet and As(III) appeared in the control samples at the end of the experiment. In Xavarido systems with added As, the oxidated inorganic species As(V) remained practically the only species in the 3 and 30 mg L^−1^ treatments, while in Eguas, the form As(V) also predominated, but As(III), MMA(V), DMA(V) and As-Bet appeared at the end of the experiment for 3 mg L^−1^, but not for 30 mg L^−1^.

The distribution of bio-adsorbed As among extracellular intracellular compartments of the biofilm is shown in Table 4. The total amount of As in these fractions was similar for both controls (0.310 and 0.276 µg g^−1^), but reached the highest value for Eguas with 30 mg L^−1^ (6.955 µg g^−1^). The extracellular As was in all cases significantly higher than the intracellular one, and increased with the As dose, reaching values of 95–96% of the total bio-adsorbed, with extracellular: intracellular ratios around 3–2 for control samples, 5–7 for 3 mg L^−1^, and 20–22 for 30 mg L^−1^, for Eguas and Xavarido, respectively. This pointed to a saturation of the intracellular absorption capacity by the organisms of the biofilm.

**Figure 6:**
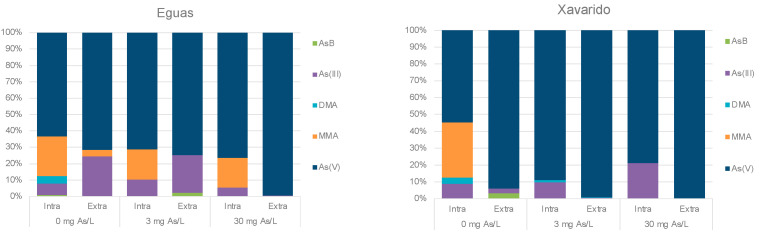
Distribution of extracellular and intracellular arsenic species found in 0, 3 and 30 mg L^−1^ systems from Eguas (**left**) and Xavarido (**right**).

Regarding As speciation in the biofilm, As(V) dominated in both compartments in all the systems studied (Figure 6). In the control samples, As(III) and MMA(V) appeared in the extracellular fraction of Eguas, and As(III) and As-Bet in Xavarido, while in the intracellular compartment of both sediments, MMA(V), DMA(V) and, in a lesser proportion As(III), coexisted with the dominant As(V). In the systems with added arsenic, As(V) still dominated in both fractions, but MMA(V) and As(III) appeared in the intracellular fraction of Eguas, and As(III) and As-Bet in the extracellular fraction, while, in Xavarido, As(III) accompanied the dominant As(V) only in the intracellular fraction.

Table 5 shows the results of the EPS analyses, estimated by the content of carbohydrates, performed in samples with 0.3, 1 and 10 mg L^−1^ of As added. The highest EPS concentrations (*p* < 0.001) were observed for the lowest As concentration in Eguas, but for the two highest in Xavarido. With respect to As concentrations in this component, they were similar between treatments in Eguas, although the As/carbohydrates ratio was higher for 10 mg L^−1^. In Xavarido, the concentration of As associated to EPS increased with added As (15 times), as did the As/carbohydrates ratio (4.5 times).

### 3.3. Development of Tolerance to As

**Figure 7:**
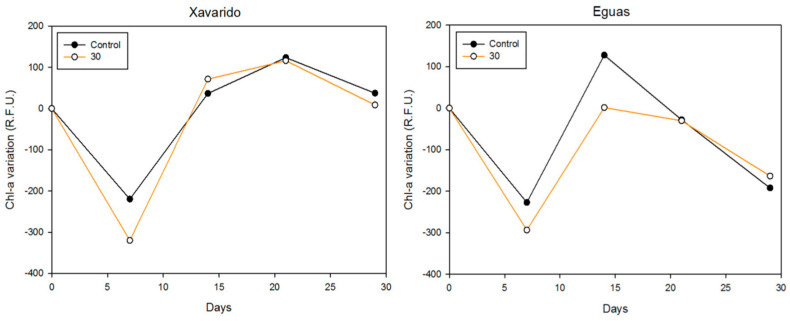
Biofilm growth in the experiment 2, measured by Chl-a fluorescence (R.F.U.).

To test the possible development of tolerance after exposition at a high As dose in the laboratory, 250 mL of a solution of 30 mg L^−1^ As were added to the previous control and 30 mg As L^−1^ systems and incubated for 29 days. Total As concentration in the overlying water during the experiment is shown in Figure 7. In the Eguas systems, the concentration in solution rapidly decreased to values < 2 mg L^−1^ from day 7, whereas in Xavarido the decrease was more gradual, reaching a final value of 15 mg L^−1^ for the previous 30-system and 9 mg L^−1^ for the previous control.

The variation (%) in Chl-a fluorescence (F_0_) with respect to the initial values changed with time, reaching a value higher than the initial one in Xavarido and lower in Eguas. There were no differences in algal growth between the previous 0 and 30 mg L^−1^ systems (Figure 7). Therefore, tolerance was not acquired by the previous exposition to added arsenic in Experiment 1. The parameters of photosynthetic capacity (Ymax) and efficiency (Yeff) confirmed this behavior, as no significant differences were observed between the previous control and 30 mg L^−1^.

## 4. Discussion

This work was designed to study the effect of As on the growth of epipsammic biofilm, and conversely, how biofilms affect the retention and transformation of this element. To this end, microcosm experiments were performed to evaluate the growth of epipsammic biofilm on riverbed sediments, with low and high natural As concentrations. The biofilms were exposed to increased concentrations of As(V), and the effects assessed, mainly focusing on autotrophic components. In all the systems evaluated, the biofilm growth was twice higher (applying the conversion factor to take account of the gain used in Phyto-PAM measures) on Xavarido sediments than on Eguas sediments, although the latter site had higher nutrient and organic matter contents, and lower natural arsenic concentrations. This pointed to a tolerance of As in naturally exposed sediments of Xavarido; additionally, the coarser texture of the Xavarido sediment (Table 2) may allow a better oxygen flow from the overlying water to the interstitial water of the sediment and, thus, better conditions for the biofilm development [41].

The results indicated that, in the range of concentrations assayed (0–30 mg L^−1^), As had no negative effect on the autotrophic components of biofilm, evaluated by in vivo Chl-a fluorescence (F_0_) (Figure 1), or in its physiological status, evaluated by means of photosynthetic capacity and efficiency (Table 3). Live/Dead analysis also revealed a high resistance to As of the bacterial community in the Xavarido biofilm, as live cell counts for the highest As concentration were even higher than in control samples (Figure 3). In a previous study on As toxicity for biofilms developed on the Anllóns riverbed sediments, their resistance to high concentrations of this toxicant and the tolerance in the Xavarido site were observed [42]. Thus, the evaluation of the acute As toxicity for epipsammic biofilms developed on Eguas and Xavarido sediments showed higher resistance for the latter, with EC20 of 0.6 and 3 mg L^−1^, respectively. An EC50 of 1 mg L^−1^ was found in Eguas, but was not reached for Xavarido samples in the range of concentrations tested (up to 100 mg L^−1^).

Other studies observed negative effects of As on biofilms at lower concentrations. In an experimental microcosm study, environmentally realistic concentrations of arsenic (15 and 130 μg L^−1^) had toxic effects on biofilms [17]. Algal growth, quality of the biofilm and contribution to nutrient cycling was affected by exposition to an average exposure concentration of 37 μg L^−1^ in [14]. Barral-Fraga et al. [43] found that exposition to 130 µg L^−1^ over 13 days reduced the cell biovolume of the diatom community and the species richness and produced a selection for tolerant species. The growth of the biofilm on complex natural sediments, instead of artificial substrates, as was the case for these studies, may have offered our research better conditions to resist the stress produced by As and, thus, fewer negative effects. Moreover, in this study, a progressive decrease in dissolved As occurred throughout the experiment. If we look at the highest concentration tested, As in water fell from 30 mg L^−1^ at the beginning to around 3 mg L^−1^ for Xavarido and 0.07 mg L^−1^ for Eguas at the end of the Experiment 1 (Figure 4). Thus, to elucidate the effect of the progressive redistribution of As in the water–biofilm–sediment system for toxicity, further research on the relative toxic effect of the As in solution or when adsorbed deserves attention.

Although an overall negative effect on biofilm growth was not observed, algal succession was indeed affected by As addition (Figure 2). This agreed with other studies where As was identified as responsible for changes in community composition (selection of most tolerant species, reduction of species richness and increase of heterotrophic character of the biofilm) and reduction of diatom cell size [14,17,43,44]. In our study, brown algae, which mostly corresponded to diatoms, were the algal group most tolerant to this element. Several authors have observed higher resistance of diatoms when exposed to As or metals [6,14,45,46,47,48,49]. Nevertheless, opposite results were obtained studying the acute effect of short exposition of the epipsammic biofilm to As [42]; in that case, diatoms were the less tolerant and green algae and cyanobacteria the most relevant. The presence of a natural sediment substrate in our case (other studies were mainly conducted on artificial inert substrates) and the different duration of the experiments might explain the differences observed.

A significant role of biofilm development on As concentrations, speciation and redistribution was clearly observed in our results. Arsenic concentration in water decreased with time during biofilm formation, and this trend was clearer for the Eguas sediments. The higher As retention in Eguas samples was likely due to their finer texture and higher organic matter content, two properties that have been indicated to positively influence As retention in sediments along the Anllóns river [50].

In the biofilm, As can be retained in three compartments: inside the cells (intracellular As), outside the cells, associated with cell walls (extracellular As) and in the EPS. The As distribution in the biofilm indicated that extracellular As was significantly higher (up to 11 times) than intracellular As, and the ratio increased with As concentration, as did the As/EPS ratios. This behavior could be attributed to limited intracellular As uptake capacity at high concentrations of As(V) in solution, so concentrations exceeding saturation tend to accumulate in the extracellular compartment. This fact has been previously described in [18], which studied the toxicity and bioaccumulation kinetics of arsenate in two freshwater algae (*Chlamydomonas reinhardtii* and *Scenedesmus obliquus*), and the bio-uptake of As species by *Chlorella salina* [51], showing that intracellular As increased linearly when As(V) concentration increased between 10 µM and 50 µM, followed by a single saturation plateau above this concentration.

The toxicity of As depends largely on its chemical form. The inorganic forms are generally considered more toxic than the organic ones [52,53]. The reduced form As(III) is generally considered more toxic and mobile than As(V) [12], due to the high affinity As(III) has for the sulfhydryl groups of biomolecules, such as glutathione, lipoic acid and the cysteinyl residues of many enzymes [53,54], altering their conformation and function, as well as their interaction with other functional proteins [55]. The methylated As(III) species are an exception to the general higher toxicity of inorganic As [56], as these organic forms are the most potent species in their direct ecotoxicological action, damaging the DNA [57].

Regarding As speciation in our experimental systems, As(V)was the predominant species in water and in the biofilm. In the aqueous phase, the products of As biotic transformation, As(III), DMA(V) and MMA(V), were also observed in control samples and for intermediate As concentrations in Eguas (Figure 5), demonstrating that the organisms in the biofilm were actively reducing As(V) to As(III), via various reductases, using glutaredoxin, glutathione or thioredoxin as an electron donor [58,59]. Methylation of As(III) is slow and it is more likely to occur in the stationary phase of algae growth [60].

In the biofilm, As(III) and methylated species were found in the control systems, which were also observed in Eguas in the presence of As. The occurrence of As(III) simultaneously with methylated As species inside the biofilm cells indicated that methylation processes were occurring intracellularly. In Xavarido, As(III) appeared inside the cells, but not the methylated species. This fact was attributed to higher As concentration in water, as it has been demonstrated that As methylation mainly occurs at low concentrations [60].

Finally, regarding the potential development of As tolerance by biofilms exposed to high As concentrations, the results showed clear evidence of native tolerance in the Xavarido biofilms, likely due to the natural high As concentrations at this site. In turn, we did not observe a tolerance acquired during exposure to As in the laboratory.

## 5. Conclusions

Arsenic concentration in solution dramatically decreased during incubation experiments, retained by sediment particles and biofilms. In the biofilm, As was mostly retained in the extracellular compartment. The biofilm affected As transformation, promoting reduction to As(III) and methylation to DMA(V) and MMA(V), in the water column and the biofilm.

Biofilms developed on sediments for all the As concentrations evaluated (0–30 mg L^−1^); therefore, a chronic toxic effect was not detected. Biofilm growth was higher on the sediments with higher As concentrations, thus pointing to acquisition of pollution-induced tolerance. However, tolerance was not acquired during exposure to high As doses (30 mg L^−1^) for 29 days in the laboratory.

These observations confirmed the relevant role that biofilms play in As biogeochemistry, by affecting its water–sediment distribution and speciation, with expected consequences in As ecotoxicology, highly affected by the availability and chemical forms of this element. The biofilm developed tolerance for As due to continuous exposure to this element and this might be one of the reasons for the differences in the toxic effects of As concentrations on biofilm constituents that were found in the literature. The compared effect of As in solution and that retained by the sediment on As toxicity to the biofilm needs further research.

This knowledge may be used for environmental purposes, mainly for the evaluation of the fate and risk of As in polluted fluvial sediments, but also for biotechnological purposes, as is the case for the design of water purification systems based on adapted biofilms that retain and transform this pollutant.

## Figures and Tables

**Table 1:** Chemical characteristics of the Anllóns River water used for the experiments.

	pH	Electrical Conductivity (µS cm^−1^)	Alkalinity (mg L^−1^)	Total N (mg L^−1^)	Total P (mg L^−1^)
Eguas	7.39	151	27.1	1.64	0.045
Xavarido	7.40	146	26.6	1.52	0.026
	Na	K	Mg	Ca	As
	(mg L^−1^)				(µg L^−1^)
Eguas	11.4	1.6	3.6	7.0	0.85
Xavarido	10.9	1.5	3.6	8.5	1.33

**Table 2:** Characterization of Anllóns River sediments.

Particle Size (%)	>2 mm	2–1 mm	1–0.5 mm	0.5–0.2 mm	0.25–0.1 mm	0.1–0.05 mm	<0.05 mm
Eguas	0.64	5.11	9.26	6.28	20.00	0.11	58.62
Xavarido	17.25	25.64	30.95	18.19	6.14	0.09	1.75
	N%	C%	S%	C/N	Al%	Fe%	Ti%
Eguas	0.60	9.19	0.13	15	6.5	6.1	0.8
Xavarido	0.06	0.44	0.01	7	6.3	4.7	1.4
	P (mg kg^−1^)	Mn (mg kg^−1^)	Ni (mg kg^−1^)	Zn (mg kg^−1^)	Cu (mg kg^−1^)	As (mg kg^−1^)	
Eguas	1349	1126	39	257	36	31	
Xavarido	n.d. ^1^	994	16	65	11	85	

^1^ not determined.

**Table 3:** Maximal quantum yield (Ymax), effective quantum yield (Yeff) and photochemical quenching (qP, based on Chl-a content) during biofilm growth in sediments exposed to different added As concentrations (mean ± SD, *n* = 3). 0 mg L^−1^ refer to control samples without added As. Natural As concentrations in the river water were 0.85 and 1.33 µg L^−1^ for Eguas and Xavarido, respectively.

			Day 0	Day 9	Day 42
Ymax	Eguas	0 mg L^−1^	0.02 ± 0.02	0.59 ± 0.02	0.49 ± 0.18
		0.3 mg L^−1^	0.02 ± 0.02	0.57 ± 0.05	0.44 ± 0.21
		1 mg L^−1^	0.01 ± 0.02	0.61 ± 0.02	0.43 ± 0.13
		3 mg L^−1^	0.04 ± 0.04	0.59 ± 0.05	0.47 ± 0.09
		10 mg L^−1^	0.04 ± 0.04	0.59 ± 0.04	0.44 ± 0.14
		30 mg L^−1^	0.05 ± 0.05	0.50 ± 0.04	0.47 ± 0.08
	Xavarido	0 mg L^−1^	0.12 ± 0.03	0.65 ± 0.01	0.52 ± 0.05
		0.3 mg L^−1^	0.13 ± 0.05	0.63 ± 0.02	0.54 ± 0.05
		1 mg L^−1^	0.09 ± 0.02	0.64 ± 0.01	0.49 ± 0.07
		3 mg L^−1^	0.12 ± 0.03	0.62 ± 0.02	0.50 ± 0.01
		10 mg L^−1^	0.09 ± 0.01	0.58 ± 0.02	0.53 ± 0.05
		30 mg L^−1^	0.12 ± 0.07	0.53 ± 0.04	0.44 ± 0.03
Yeff	Eguas	0 mg L^−1^	0.17 ± 0.06	0.54 ± 0.06	0.58 ± 0.01
		0.3 mg L^−1^	0.04 ± 0.01	0.55 ± 0.04	0.39 ± 0.18
		1 mg L^−1^	0.10 ± 0.07	0.53 ± 0.04	0.59 ± 0.01
		3 mg L^−1^	0.06 ± 0.02	0.54 ± 0.03	0.50 ± 0.05
		10 mg L^−1^	-	0.43 ± 0.03	0.51 ± 0.02
		30 mg L^−1^	0.03 ± 0.01	0.39 ± 0.05	0.55 ± 0.02
	Xavarido	0 mg L^−1^	0.06 ± 0.06	0.48 ± 0.07	0.49 ± 0.03
		0.3 mg L^−1^	0.12 ± 0.00	0.51 ± 0.02	0.46 ± 0.08
		1 mg L^−1^	0.08 ± 0.09	0.57 ± 0.04	0.39 ± 0.08
		3 mg L^−1^	0.03 ± 0.05	0.53 ± 0.05	0.43 ± 0.11
		10 mg L^−1^	0.04 ± 0.01	0.47 ± 0.02	0.46 ± 0.03
		30 mg L^−1^	0.16 ± 0.17	0.47 ± 0.15	0.39 ± 0.01
qP	Eguas	0 mg L^−1^	0.62 ± 0.48	0.90 ± 0.03	0.94 ± 0.01
		0.3 mg L^−1^	0.32 ± 0.23	0.94 ± 0.04	0.86 ± 0.12
		1 mg L^−1^	−1.31 ± 3.63	0.88 ± 0.04	0.96 ± 0.05
		3 mg L^−1^	0.07 ± 0.64	0.92 ± 0.09	0.93 ± 0.03
		10 mg L^−1^	−0.74 ± 0.77	0.88 ± 0.04	0.92 ± 0.04
		30 mg L^−1^	−0.05 ± 0.64	0.99 ± 0.07	0.91 ± 0.06
	Xavarido	0 mg L^−1^	0.82 ± 0.98	0.84 ± 0.07	0.85 ± 0.02
		0.3 mg L^−1^	6.56 ± 9.56	0.86 ± 0.10	0.78 ± 0.14
		1 mg L^−1^	0.83 ± 0.61	0.89 ± 0.06	0.75 ± 0.11
		3 mg L^−1^	3.75 ± 2.17	0.87 ± 0.06	0.81 ± 0.15
		10 mg L^−1^	0.69 ± 0.27	0.80 ± 0.05	0.85 ± 0.01
		30 mg L^−1^	3.40 ± 3.38	0.91 ± 0.09	0.80 ± 0.01

**Table 4:** Total extracellular and intracellular As (mg kg^−1^) at the end of Experiment 1 in Eguas and Xavarido systems (mean ± SD, *n* = 3).

		Extracellular As	Intracellular As
Eguas	Control	0.230 ± 0.015	0.080 ± 0.007
	3 mg L^−1^	0.400 ± 0.049	0.083 ± 0.033
	30 mg L^−1^	6.630 ± 1.669	0.325 ± 0.249
Xavarido	Control	0.182 ± 0.018	0.093 ± 0.008
	3 mg L^−1^	1.571 ± 0.197	0.218 ± 0.048
	30 mg L^−1^	2.097 ± 0.527	0.096 ± 0.031

**Table 5:** Total carbohydrates and As in EPS in the biofilm from Eguas and Xavarido (mg g^−1^ dry sediment); (mean ± SD, *n* = 3).

	As	Carbohydrates (mg g^−1^)	As EPS (µg g^−1^)	As/Carbohydrates
Eguas	0.3 mg L^−1^	0.62 ± 0.11	0.30 ± 0.06	0.5
	1 mg L^−1^	0.37 ± 0.13	0.20 ± 0.10	0.5
	10 mg L^−1^	0.38 ± 0.28	0.34 ± 0.32	0.9
Xavarido	0.3 mg L^−1^	0.24 ± 0.03	0.09 ± 0.03	0.4
	1 mg L^−1^	0.60 ± 0.14	0.62 ± 0.07	1.0
	10 mg L^−1^	0.76 ± 0.16	1.36 ± 0.09	1.8

## Data Availability

Not applicable.

## Supplementary Materials

The following supporting information can be downloaded at: https://www.mdpi.com/article/10.3390/ijerph191912689/s1, Table S1: Maximal quantum yield (Ymax, based on Chl-a content), effective quantum yield (Yeff, based on Chl-a content) and photochemical quenching (qP, based on Chl-a content) during biofilm growth in sediments exposed to different As concentrations; Table S2: Biofilm algae distribution (%) for the different As concentrations B1: blue algae, Gr: green algae and Br: brown algae.
