# Supplementary material for: Assessment of the Chronic Toxicity and Interactions between Arsenic and Riverbed Biofilms
Source: Int J Environ Res Public Health. 2022 Oct 4;19(19):12689. doi: 10.3390/ijerph191912689 (PMC9564892; doi:10.3390/ijerph191912689)
Supplement: Supplementary file 1 [file ijerph-19-12689-s001.zip › ijerph-1865458-supplementary.pdf]

Supplementary material.

Table S1. Maximal quantum yield (Ymax, based on Chl-a content), effective quantum yield (Yeff, based on Chl-a content) and photochemical quenching (qP, based on Chl-a content) during biofilm growth in sediments exposed to different As concentrations.

Ymax		Day						
Eguas		0	9	14	22	28	35	42
	0 mg L^{-1}	0.02 ± 0.02	0.59 ± 0.02	0.63 ± 0.00	0.50 ± 0.06	0.50 ± 0.06	0.49 ± 0.08	0.49 ± 0.18
	0.3 mg L^{-1}	0.02 ± 0.02	0.57 ± 0.05	0.59 ± 0.09	0.51 ± 0.05	0.55 ± 0.10	0.51 ± 0.12	0.44 ± 0.21
	1 mg L^{-1}	0.01 ± 0.02	0.61 ± 0.02	0.64 ± 0.01	0.48 ± 0.12	0.49 ± 0.19	0.49 ± 0.14	0.43 ± 0.13
	3 mg L^{-1}	0.04 ± 0.04	0.59 ± 0.05	0.61 ± 0.01	0.50 ± 0.06	0.49 ± 0.10	0.48 ± 0.08	0.47 ± 0.09
	10 mg L	-	0.59 ± 0.04	0.61 ± 0.03	0.51 ± 0.04	0.43 ± 0.08	0.31 ± 0.08	0.44 ± 0.14
	30 mg L^{-1}	0.05 ± 0.05	0.50 ± 0.04	0.61 ± 0.01	0.51 ± 0.03	0.42 ± 0.01	0.36 ± 0.11	0.47 ± 0.08
Xavarido		0	9	14	22	28	35	42
	0 mg L^{-1}	0.12 ± 0.03	0.65 ± 0.01	0.62 ± 0.02	0.62 ± 0.01	0.60 ± 0.02	0.59 ± 0.03	0.52 ± 0.05
	0.3 mg L^{-1}	0.13 ± 0.05	0.63 ± 0.02	0.60 ± 0.03	0.57 ± 0.03	0.55 ± 0.03	0.53 ± 0.05	0.54 ± 0.05
	1 mg L^{-1}	0.09 ± 0.02	0.64 ± 0.01	0.54 ± 0.07	0.46 ± 0.14	0.46 ± 0.07	0.48 ± 0.02	0.49 ± 0.07
	3 mg L^{-1}	0.12 ± 0.03	0.62 ± 0.02	0.56 ± 0.03	0.48 ± 0.03	0.47 ± 0.07	0.48 ± 0.02	0.50 ± 0.01
	10 mg L	0.09 ± 0.01	0.58 ± 0.02	0.61 ± 0.07	0.52 ± 0.04	0.49 ± 0.05	0.52 ± 0.07	0.53 ± 0.05
	30 mg L^{-1}	0.12 ± 0.07	0.53 ± 0.04	0.56 ± 0.11	0.49 ± 0.01	0.49 ± 0.09	0.46 ± 0.08	0.44 ± 0.03
Yeff								
Eguas		0	9	14	22	28	35	42
	0 mg L^{-1}	0.17 ± 0.06	0.54 ± 0.06	0.57 ± 0.01	0.42 ± 0.05	0.45 ± 0.02	0.59 ± 0.01	0.58 ± 0.01
	0.3 mg L^{-1}	0.04 ± 0.01	0.55 ± 0.04	0.59 ± 0.06	0.45 ± 0.07	0.35 ± 0.21	0.42 ± 0.20	0.39 ± 0.18
	1 mg L^{-1}	0.10 ± 0.07	0.53 ± 0.04	0.58 ± 0.00	0.51 ± 0.07	0.59 ± 0.03	0.57 ± 0.02	0.59 ± 0.01
	3 mg L^{-1}	0.06 ± 0.02	0.54 ± 0.03	0.58 ± 0.01	0.48 ± 0.05	0.41 ± 0.16	0.26 ± 0.29	0.50 ± 0.05
	10 mg L	-	0.43 ± 0.03	0.56 ± 0.02	0.49 ± 0.06	0.59 ± 0.00	0.51 ± 0.00	0.51 ± 0.02
	30 mg L^{-1}	0.03 ± 0.01	0.39 ± 0.05	0.40 ± 0.09	0.38 ± 0.24	0.42 ± 0.17	0.41 ± 0.04	0.55 ± 0.02
Xavarido		0	9	14	22	28	35	42
	0 mg L^{-1}	0.06 ± 0.06	0.48 ± 0.07	0.41 ± 0.02	0.50 ± 0.03	0.45 ± 0.06	0.52 ± 0.03	0.49 ± 0.03

	0.3 mg L^{-1}	0.12 ± 0.00	0.51 ± 0.02	0.41 ± 0.18	0.53 ± 0.09	0.48 ± 0.02	0.50 ± 0.02	0.46 ± 0.08
	1 mg L^{-1}	0.08 ± 0.09	0.57 ± 0.04	0.30 ± 0.26	0.21 ± 0.15	0.33 ± 0.28	0.35 ± 0.15	0.39 ± 0.08
	3 mg L^{-1}	0.03 ± 0.05	0.53 ± 0.05	0.30 ± 0.08	0.34 ± 0.19	0.27 ± 0.05	0.34 ± 0.11	0.43 ± 0.11
	10 mg L	0.04 ± 0.01	0.47 ± 0.02	0.35 ± 0.30	0.34 ± 0.11	0.47 ± 0.04	0.46 ± 0.08	0.46 ± 0.03
	30 mg L^{-1}	0.16 ± 0.17	0.47 ± 0.15	0.30 ± 0.33	0.26 ± 0.37	0.26 ± 0.36	0.25 ± 0.35	0.39 ± 0.01
qP								
Eguas		0	9	14	22	28	35	42
	0 mg L^{-1}	0.62 ± 0.48	0.90 ± 0.03	0.93 ± 0.03	0.83 ± 0.02	0.72 ± 0.13	0.93 ± 0.03	0.94 ± 0.01
	0.3 mg L^{-1}	0.32 ± 0.23	0.94 ± 0.04	0.96 ± 0.05	0.84 ± 0.08	0.75 ± 0.19	0.79 ± 0.15	0.86 ± 0.12
	1 mg L^{-1}	-1.31 ± 3.63	0.88 ± 0.04	0.94 ± 0.02	0.66 ± 0.49	1.00 ± 0.11	0.77 ± 0.24	0.96 ± 0.05
	3 mg L^{-1}	0.07 ± 0.64	0.92 ± 0.09	0.95 ± 0.01	0.75 ± 0.13	0.86 ± 0.07	0.44 ± 0.41	0.93 ± 0.03
	10 mg L	-0.74 ± 0.77	0.88 ± 0.04	0.96 ± 0.07	0.87 ± 0.03	0.32 ± 0.53	0.61 ± 0.54	0.92 ± 0.04
	30 mg L^{-1}	-0.05 ± 0.64	0.99 ± 0.07	0.90 ± 0.05	0.60 ± 0.31	0.83 ± 0.12	0.71 ± 0.09	0.91 ± 0.06
Xavarido		0	9	14	22	28	35	42
	0 mg L^{-1}	0.82 ± 0.98	0.84 ± 0.07	0.54 ± 0.40	0.84 ± 0.08	0.84 ± 0.06	0.87 ± 0.06	0.85 ± 0.02
	0.3 mg L^{-1}	6.56 ± 9.56	0.86 ± 0.10	0.73 ± 0.15	0.85 ± 0.07	0.74 ± 0.12	0.88 ± 0.01	0.78 ± 0.14
	1 mg L^{-1}	0.83 ± 0.61	0.89 ± 0.06	0.52 ± 0.44	0.52 ± 0.32	0.57 ± 0.49	0.79 ± 0.21	0.75 ± 0.11
	3 mg L^{-1}	3.75 ± 2.17	0.87 ± 0.06	0.62 ± 0.10	0.68 ± 0.17	0.65 ± 0.10	0.72 ± 0.07	0.81 ± 0.15
	10 mg L	0.69 ± 0.27	0.80 ± 0.05	0.85 ± 0.08	0.65 ± 0.09	0.81 ± 0.10	0.82 ± 0.06	0.85 ± 0.01
	30 mg L^{-1}	3.40 ± 3.38	0.91 ± 0.09	0.99 ± 0.09	0.29 ± 0.49	0.63 ± 0.55	0.53 ± 0.46	0.80 ± 0.01

Table S2. Biofilm algae distribution (%) for the different Arsenic concentrations B1: blue algae, Gr: green algae and Br: brown algae.

**Eguas**	**DAY 0**			**DAY 4**			**DAY 9**			**DAY 14**			**DAY 22**			**DAY 28**			**DAY 35**			**DAY 42**		
As mg/L	**Bl**	**Gr**	**Br**	**Bl**	**Gr**	**Br**	**Bl**	**Gr**	**Br**	**Bl**	**Gr**	**Br**	**Bl**	**Gr**	**Br**	**Bl**	**Gr**	**Br**	**Bl**	**Gr**	**Br**	**Bl**	**Gr**	**Br**
0	3.5	12.7	83.8	19.2	15.6	65.2	58.8	0.7	40.5	77.2	0.1	22.6	57.1	0.9	42.0	46.7	6.2	47.0	35.7	15.8	48.5	65.6	1.0	33.4
0,3	0.0	10.4	89.6	27.8	15.0	57.1	56.4	1.2	42.3	66.3	2.0	31.7	59.9	5.7	34.4	37.6	19.2	43.1	33.3	17.6	49.1	44.4	7.4	48.2
1	0.0	17.3	82.7	35.0	9.0	56.0	71.1	0.4	28.5	58.1	0.9	41.0	52.9	4.5	42.6	47.5	2.3	50.2	42.4	5.9	51.8	53.5	5.5	41.0
3	0.0	23.4	76.6	31.7	5.0	63.4	54.4	0.0	45.6	62.5	1.8	35.7	51.1	2.9	46.0	34.7	8.6	56.8	32.2	16.8	51.0	36.4	8.5	55.1
10	0.0	20.2	79.8	33.7	10.8	55.6	60.9	0.7	38.4	62.3	0.5	37.2	63.2	1.0	35.8	44.4	3.1	52.4	31.8	2.4	65.8	47.4	0.5	52.2
30	0.0	18.9	81.1	24.0	30.0	46.0	54.6	7.4	38.0	49.1	0.4	50.6	55.0	0.4	44.7	33.0	11.0	56.0	28.3	10.7	61.0	36.2	16.6	47.1
**Xavarido**	**DAY 0**			**DAY 4**			**DAY 9**			**DAY 14**			**DAY 22**			**DAY 28**			**DAY 35**			**DAY 42**		
As mg/L	**Bl**	**Gr**	**Br**	**Bl**	**Gr**	**Br**	**Bl**	**Gr**	**Br**	**Bl**	**Gr**	**Br**	**Bl**	**Gr**	**Br**	**Bl**	**Gr**	**Br**	**Bl**	**Gr**	**Br**	**Bl**	**Gr**	**Br**
0	32.6	10.1	57.3	38.7	10.0	51.3	27.0	0.0	73.0	28.3	0.0	71.7	33.4	0.0	66.6	34.9	0.9	64.2	45.5	9.0	45.5	74.8	0.0	25.2
0,3	28.0	12.7	59.3	37.7	3.7	58.6	30.8	0.0	69.2	25.0	3.6	71.4	28.3	6.7	65.0	33.8	10.9	55.2	42.7	5.0	52.3	53.9	3.0	43.1
1	27.7	16.5	55.7	37.3	7.8	54.9	27.3	0.0	72.7	22.9	1.3	75.8	25.9	0.9	73.2	28.7	2.6	68.7	42.2	7.6	50.2	52.0	0.0	48.0
3	23.9	20.5	55.7	36.5	6.7	56.8	32.2	0.0	67.8	22.5	1.7	75.9	25.4	5.5	69.1	28.2	12.7	59.1	37.2	12.3	50.5	48.5	0.8	50.7
10	24.7	23.7	51.6	43.1	10.1	46.8	34.9	0.0	65.1	22.3	0.0	77.7	25.0	2.3	72.7	27.3	5.7	67.0	31.8	10.8	57.4	50.0	1.5	48.5
30	25.1	24.3	50.6	40.6	8.5	50.8	35.1	0.0	64.9	23.4	0.1	76.5	22.3	5.4	72.2	25.9	6.1	68.0	29.1	5.3	65.5	42.4	0.3	57.3
